# Tameness correlates with domestication related traits in a Red Junglefowl intercross

**DOI:** 10.1111/gbb.12704

**Published:** 2020-10-12

**Authors:** Rebecca Katajamaa, Per Jensen

**Affiliations:** ^1^ IFM Biology Linköping University Linköping Sweden

**Keywords:** chickens, correlated selection responses, domestication, fear behaviour, tameness

## Abstract

Early animal domestication may have been driven by selection on tameness. Selection on only tameness can bring about correlated selection responses in other traits, not intentionally selected upon, which may be one cause of the domesticated phenotype. We predicted that genetically reduced fear towards humans in Red Junglefowl, ancestors of domesticated chickens, would be correlated to other traits included in the domesticated phenotype. Fear level was determined by a standardised behaviour test, where the reaction towards an approaching human was recorded. We first selected birds for eight generations for either high or low fear levels in this test, to create two divergent selection lines. An F3 intercross, with birds from the eighth generation as parentals, was generated to study correlations between fear‐of‐human scores and other unselected phenotypes, possibly caused by pleiotropy or linkage. Low fear‐of‐human scores were associated with higher body weight and growth rates, and with increased activity in an open field test, indicating less general fearfulness. In females, low fear‐of‐human scores were also associated with more efficient fear habituation and in males with an increased tendency to emit food calls in a mirror test, indicating increased social dominance. Low fear‐of‐human scores were also associated with smaller brain relative to body weight, and with larger cerebrum relative to total brain weight in females. All these effects are in line with the changes observed in domesticated chickens compared to their ancestors, and we conclude that tameness may have been a driving factor underlying some aspects of the domesticated phenotype.

## Graphical abstract

Fear‐of‐human score correlates to domestication related phenotypes in a Red Junglefowl intercross.

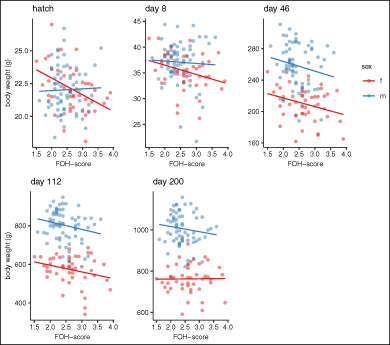

## 1 INTRODUCTION

Animal domestication has been defined as a process in which humans are responsible for the selection of traits that cause changes in a population,^1^ as such it is a special case of evolution driven by humans. However, natural selection still plays a relevant role.^2^ Individuals that cannot cope with the captive environment are not likely to thrive, regardless of human intervention. Consequently, early domestication in animals is thought to have been initially driven largely by selection on tameness.^3^, ^4^ An interesting aspect of selection for tameness, whether done consciously or not, is that other traits that are not specifically selected upon can change due to being genetically correlated. This may produce some of the phenotypic traits that are commonly termed “the domesticated phenotype”, including, for example, changes in body size, loss of pigmentation and increased reproductive capacity.^5^ Experiments where animals have been selected for tameness have been done in several species, e.g. silver foxes,^6^ rats,^7^ mink^8^ and chickens.^9^ From these studies, it is clear that some correlated selection responses indeed are consistent with the evolution of the domesticated phenotype. For example, selected only for reduced fear of humans, silver foxes started to resemble domesticated dogs with, among other traits, floppy ears, white spots and characteristic whimpering for human attention after only a few generations.^6^

Chickens were domesticated at least 8000 years ago in India and South‐East Asia.^10^ By selecting on tameness in ancestral Red Junglefowl that can still be found in the wild, we have been able to model a possible early domestication scenario in a species that has already been domesticated once. For many of our domesticated animal species, there are no extant ancestors available, which make this a unique study in its own right. We have found that when selecting for reduced fear of humans only, birds developed a variety of traits consistent with the domesticated phenotype within few generations,^4^ for example increased body size and reproductive output, reduced relative brain size and increased social dominance.^9^, ^11^

In order to explore the possible genetic underpinnings of correlated selection responses to tameness, and thereby to shed light on the mechanisms by which reduced fear of humans affect the domesticated phenotype, we generated an intercross line between the two selection lines. In an intercross line, traits that are affected by the same genes or by genes located closely together on a chromosome will segregate together. Hence, correlations between traits in such a setup means they are likely to be affected by similar genetic mechanisms, given that all other conditions are similar.^12^ This can be caused by mainly two genetic processes. Pleiotropy is the situation in which two traits that seem to be unrelated are determined by the same locus.^13^ Linkage, on the other hand, is when two separate loci are inherited together because they are situated close to each other on a chromosome. For quantitative traits, such as body weight and most behaviours, genetic resolution is usually poor as they are most often determined by many different genes exerting small effects, and therefore, an advanced intercross line can be generated to use recombination to increase the chance of detecting common genetic mechanisms.^14^ Similar methods have previously been used to assess the genetic contributions to phenotypic differences between Red Junglefowl and domesticated White Leghorn layers.^15^

The aim of this paper was to study if level of fear of humans in an F3 intercross between two Red Junglefowl lines selected for diverging levels of tameness correlates with other phenotypes. Based on previous studies of our selected Red Junglefowl lines, we predicted that birds with lower FOH‐scores towards humans would be larger and have relatively smaller brains. Furthermore, we predicted that birds with low scores for fear of humans would also habituate faster to fearful stimuli and show less fearful behaviour in an open field test. Furthermore, we predicted that they would show evidence of social dominance in a mirror test.

## 2 MATERIAL AND METHODS

### 2.1 Animals and housing

We used a third generation intercross between Red Junglefowl that had been selected for high and low fear of humans for eight generations. A detailed description of the breeding of the selection lines as well as the intercross will be given below. The housing routines were the same for all of the animals. Upon hatching, the chicks were weighed and wing tagged individually. They were housed in one group in a pen measuring 1.5 × 1.5 m for the first 1–3 weeks with heat lamps after which the pen was expanded to 1.5 × 2.25 m, heat lamps were removed and perches were added. The floor was littered with wood shavings and the chicks had ad libitum access to feed and water. At 5 weeks of age, the chickens were moved to another housing facility into three‐level aviary pens (3 × 3 × 3 m) furnished with perches and nest boxes as well as access to an outdoor aviary (3 × 3 × 3 m) and ad libitum access to feed and water. A 12:12 h dark: light cycle was maintained in both housing facilities. Feed and water were provided in multiple locations and levels to ensure that all animals had free access to the feed.

#### 2.1.1 Breeding of selection lines

Two different zoo populations, Copenhagen zoo and Götala research station (see^16^ for more information on the background of these populations), were the origin of our selection lines. These zoo populations were first interbred for two generations to create an outbred parental generation for the subsequent selection lines. As the starting point of our selection lines, we separately bred the least and most fearful individuals among the outbred parents. Fearfulness towards humans, fear‐of‐human (FOH) score, was determined individually in all animals using a standardised behaviour test (described in more detail below). Selection on FOH in our two selection lines was done for eight generations before creating an intercross line. More information about the breeding programme for the selection lines, including population sizes, can be found in.^9^

#### 2.1.2 Breeding of the intercross line

In order to study possible correlations between FOH and the phenotypes previously found to differ in our selection lines, we created an advanced intercross line. Four individuals (two males and two females) from each selection line, in total eight individuals, from the eighth selected generation were used to create an intercross line, generation F1. Each breeding pair consisted of one individual from each selection line, with reciprocal design. In the F1 and F2 generations, birds were randomly bred to generate an F3 generation intercross. Out of 34 individuals hatched in the F1 generation, six females and six males were randomly selected to create breeding pairs. Out of a total of 76 individuals in the F2 generation, 19 females and 19 males were randomly assigned into breeding pairs. In total, 134 individuals were hatched in the F3 generation. All birds in the different generations were tested in the fear of human test (see below), although FOH scores obtained from the test were not a determining factor in the choice of breeding animals as those were randomly chosen from F1 and F2 birds. The advantage of using an advanced intercross line, which is breeding past the F2 generation, is that the number of recombination events increase, and hence shortens the length of the haplotype blocks, which increases the precision in finding genetic correlations between quantitative traits such as behaviours.^12^ We do not have any information on the recombination rates in the present cross, and since we therefore do not know the average haplotype block size, it is impossible to differentiate between linkage and pleiotropy. However, the average recombination rates in chickens have been estimated to vary between 2.5 and 21 cM per Mb, being higher on microchromosomes than on macrochromosomes.^17^

### 2.2 Fear of human test

A standardised behavioural test was used to determine fearfulness towards humans when the birds were 12 weeks old. This was done for all birds in all generations and was identical to the test used in breeding the selection lines. For the F3 generation, we tested 47 females and 66 males. The test has been described in detail previously.^9^ Each bird was tested individually in a longitudinal arena (measuring 100 × 300 × 210 cm), in which the reaction to an approaching human was recorded. The arena was covered with wire mesh and from the ground up to 50 cm there was an opaque screen. Three equally sized zones (100 × 100 cm) were defined. Before the start of the test, a bird was placed on its own in the middle zone of the arena, while the arena was kept in darkness. The observer, who at the same time was the human stimulus, entered in one of the peripheral zones and remained still for 60 s. The observer always wore the same clothing. The starting point of the test was determined by the lights being turned on and at this point observations were begun. Every 10 s during a total of 180 s, the fear level of the chicken was recorded according to a 5‐point scale (Table 1), where score 1 is least fearful and score 5 is most fearful reaction. After 60 s, the observer moved to the middle zone and after 120 s, the observer moved to the last zone, adopting the same upright and motionless posture. At the end of the test (after 180 s), the observer attempted to touch the chicken without chasing it, and noted the fear reaction to that as well using the same score. The overall FOH score for each individual was determined as the mean of all the sampling points of the test.

**TABLE 1:** Ethogram describing the different fear reaction levels identified in the fear of human (FOH) (from Agnvall et al^9^) and fear habituation tests, and the behaviours recorded in the mirror test

FOH‐score in Fear of human test	Definition
1	Exploring, standing or walking, with short neck.
2	Standing or walking with eyes open and neck stretched. Head flicks and vocalising 1–5/10 sec.
3	Standing or walking with eyes open and neck stretched. Head flicks and vocalising 6–15/10 sec.
4	Standing or walking with eyes open and neck stretched. Head flicks and vocalising >15/10 sec.
5	Escape attempts and vocalising loudly alt. The bird is completely still (freeze behaviour).
**Fear reaction in fear habituation test**	**Definition**
1	No reaction.
2	Mild reaction; turns head, takes a few steps, no rushed movements.
3	Startled; Runs/walks fast one length of arena, reaction delayed ~1 sec. Escape attempt. None or short duration of wing flapping. More than 1 length of arena if not hitting wall.
4	Very startled; wing flapping and running into walls. Moves 2 or more lengths of arena. Stops briefly when hitting wall before continuing.
5	Extremely startled; rapid, prolonged wing flapping, repeatedly running into walls, 3 or more lengths of the arena moved.
6	Freeze
**Behaviour in mirror test**	**Definition**
Latency to food zone	Time from start of test to reach food zone (< half body length from food container).
Latency to food peck	Time from start of test to first food peck (any kind) in container.
Latency to food call	Time from start of test until food call is produced, males only.
Food call	Short clucks emitted when finding food, males only.

### 2.3 Body weight

The chickens were weighed at hatch, and thereafter when they were eight, 46, 112 and 200 days old using a scale with 0.01 g precision. From hatch to day 112, 47 females and 66 males were weighed. At day 200, due to mortality, only 46 females and 63 males remained for weighing. We also calculated the weight gain between weighing days as a measure of growth rate.

### 2.4 Brain tissue collection

When the birds were 32 weeks old, we weighed all birds still alive (46 females and 59 males), then culled them by rapid decapitation and performed a four‐piece dissection of the brain directly after culling according to the protocol used by Henriksen et al.^18^ The brain was divided into the cerebral hemispheres, optic lobes, a brainstem region and the cerebellum. Each individual brain region was weighed directly after dissection (wet mass) using a scale with 0.001 g precision.

### 2.5 Fear habituation test

As chicks usually show a strong behavioural response to sudden flashes of light, the birds were tested for their ability to habituate to repeated light flashes using a test where we previously found faster habituation in the low fear selection lines (Katajamaa and Jensen, in press). Chicks from the low fear line habituate quicker by showing a milder behavioural response on the second day of exposure. The test was performed in 49 females and 66 males when they were 8 days old. The arena (25 × 25 × 30 cm) had a blue LED light (round light source, 48 mm diameter) at the bottom and the floor was covered by a transparent corrugated plastic film to avoid slippage.

The test was run over two consecutive days (with 24–28 h between tests) in which the birds were exposed to five consecutive blue light flashes on each day. Before the start of the test, the chicks were placed in the arena with the lights turned off. Thirty seconds after the lights had been turned on, the chicks were exposed to the first light flash. Each light flash was then separated by 30 s until a total of five light flashes, each lasting 1 s, had been reached. The chicks were removed 30 s after the last light flash. The procedure was the same on both days.

Analysis was done from video recordings of the test sessions. The fear reaction towards each of the light flashes was determined by a pre‐defined scale (Table 1). The first author did all the behaviour recordings, and a random selection of 10% of the videos were re‐sampled by the last author to check the consistency of the recordings. Observer score correlation was r_S_ = 0.910, *p* < 0.001.

### 2.6 Open field test

To assess general fearfulness and exploration, the chicks (45 females, 63 males) were tested in an open field test when they were 4 weeks old. A previous study of the selection lines found that chicks from the low fear line were bolder in the open field test, showing a higher locomotory activity.^9^ The arena used (80 × 120 × 40 cm) was divided into two sections, centre zone (40 × 80 cm) and periphery outside of the centre zone. We also used a start zone (20 × 20) in the corner where the chicks were introduced into the arena. Each chick was placed individually in the arena with the lights turned off. Once the lights had been turned on, the chicks were allowed to freely explore the arena for 5 min before being removed and returned to the home pen. EthoVision (Noldus, version XT 10) was used for automatic recording of the movements in the arena. The test was replicated twice on two consecutive days (24–28 h in between). Total distance moved and time spent in the centre was recorded. The averages from the two test sessions were used for the statistical analysis.

### 2.7 Mirror test

When the birds were 22 weeks old, the birds were exposed to a mirror test to measure the behavioural reaction in a food competition situation involving a mirror image that we assume the chickens perceive as an unknown individual of the same sex as they do respond to it.^19^ We tested 45 females and 63 males. Previous studies have shown that birds from the low fear line are more socially dominant.^11^, ^20^ In order to assess social behaviour on an individual level, which was necessary to be able to correlate it with the FOH‐score, we substituted the social competitors with a mirror to act as an unknown social opponent. An arena (95 × 95 × 180) with a mirror (22 × 35 cm) on one side was used. A small, half‐moon‐shaped, green cup (6.5 cm wide, 4 cm high, radius = 5 cm) filled with canned corn was placed immediately in front of the mirror. The top of the arena was covered with a metal grid to avoid escape and wood shavings were used to cover the floor. A spotlight was attached to the metal grid covering the arena to provide extra lighting.

Before the test, each bird was allowed 180 s in the arena for habituation. A partition (75 × 58 cm) connected to a string was covering the mirror and cup of canned corn during the habituation. At the start of the test, the partition was lifted to reveal the mirror and cup containing canned corn. Once the partition had been lifted, the animal was left in the arena for a further 180 s. The tests were done in the afternoon not to interfere with egg laying in the females. A video camera (GoPro model Hero5) was placed at ground level in one of the corners opposite of the side where the mirror was positioned. Behaviours (Table 1) were observed using continuous recording from the videos after the test. The birds had been fed with canned corn for three consecutive days prior to the test to make them accustomed to this otherwise novel, but highly preferred, food source, but they had no prior experience of mirrors.

### 2.8 Statistical methods

All variables related to body size, brain size, fear habituation test and open field test.

were tested with a generalised linear model using SPSS version 26. Scale response linear was used in all cases where there was a normal distribution of the data. The variables measured in the open field‐ and mirror tests were not normally distributed and where therefore tested with gamma log link instead. For the mirror test, the variables crowing frequency and frequency of food calls produced by males were tested with a spearman rank correlation test instead due to the distribution of the data from these two behaviours. FOH‐score and sex were used as main effects for all tests, except for brain size measurements, and we tested the interaction between them. Average FOH‐score for males emitting food calls in the mirror test was tested against the average for the rest using a *t* test.

In order to test the differences in brain size between the sexes, we performed a *t* test for all absolute brain measurements. There were large sex differences in brain size, and due to this we tested the brain size measurements separately for the sexes. When testing the associations between brain size and FOH‐score we again used generalised linear models. When testing effects of absolute brain size, body weight was added as a covariate for total brain mass and ROB was used as a covariate in the analysis of each separate brain region. ROB was calculated for each region specifically by subtracting the region weight from the total brain weight.

To analyse fear habituation, the observed fear reaction to the light flash was plotted against exposure number^1^, ^2^, ^3^, ^4^, ^5^ for each individual, and the area under the curve (AUC) was used as a measure of the degree of habituation. AUC is a common method that integrates the responses of an individual over time and provides an overall picture of the intensity and duration of the reactions (see for example^21^).

## 3 RESULTS

### 3.1 Behaviour tests

**FIGURE 1:**
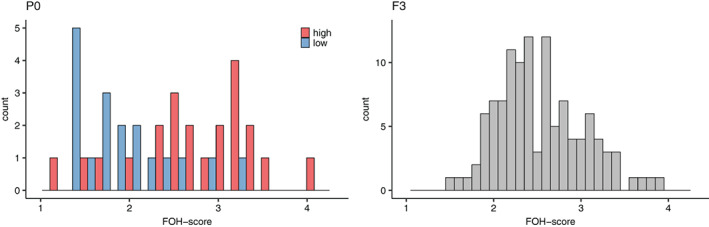
Distribution of the fear of human (FOH) test scores in the Red Junglefowl from the parental animals in the eighth generation selected for diverging levels of fear towards humans and the F3 intercross

The distribution of FOH‐scores in the parental generation (eighth generation selected for divergent levels of fear) was clearly bimodal as expected, while it was almost normal in the F3 as a result of the intercrossing (Figure 1). In the parental generation, the FOH‐scores of the two selection lines were significantly different from each other with higher scores in the high fear line compared to the low fear line (2.77 ± 0.15 versus 1.91 ± 0.13 [mean ± SE]; t = 4.311, *p* < 0.001). In the F3 intercross, the same score was intermediate to that of the parentals with an expected normal distribution (2.53 ± 0.046 [mean ± SE]).

**FIGURE 2:**
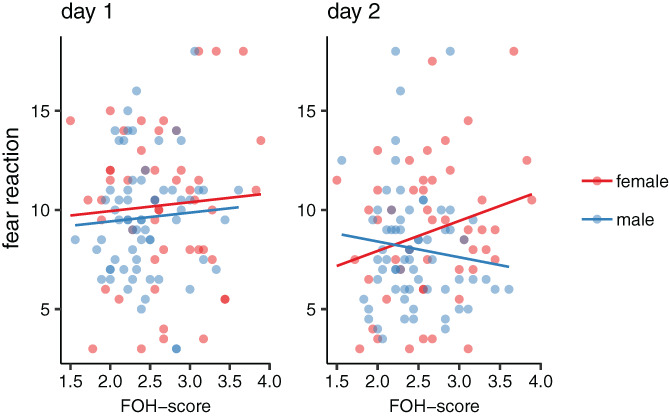
Scatterplots of fear reactions (area under the curve) in the fear habituation test plotted against fear‐of‐human (FOH) score in males and females

In the fear habituation test, there were no effects of FOH‐score on the fear reaction on day one (Supplementary Table S4). However, on day two, there was an interaction effect between FOH‐ score and sex (χ^2^_1_ = 4.306, *p* = 0.038). Lower FOH‐scores were associated with weaker fear reactions, hence a more efficient habituation, in females in the test (Figure 2).

**FIGURE 3:**
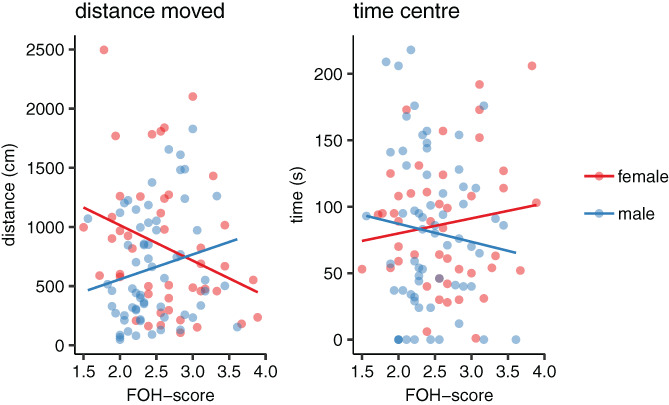
Scatterplots of variables recorded in open field test plotted against fear‐of‐human (FOH) score in males and females

Distance moved in the open field test was affected by an interaction effect between FOH‐score and sex (χ^2^_1_ = 6.003, *p* = 0.014), again with females with lower FOH‐scores moving longer distances in the test whereas males with lower FOH‐scores moved shorter distances (Figure 3).

**FIGURE 4:**
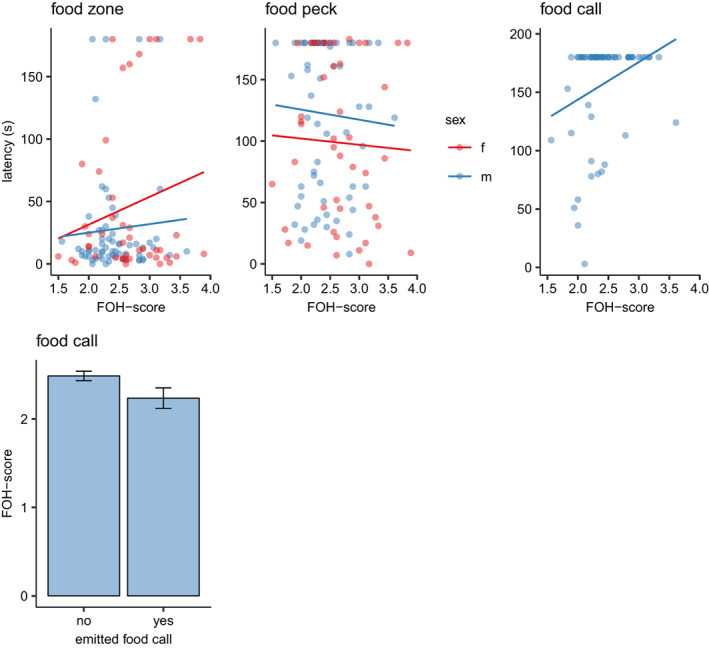
Scatterplots of variables recorded in the mirror test plotted against fear‐of‐human (FOH) score in males and females, and the average FOH‐score (+/− SEM) of males emitting at least one food call compared to males not emitting any food calls during the test

In the mirror test, there was a tendency for males with lower FOH‐score to emit food calls sooner than those with higher scores (χ^2^_1_ = 2.708, *p* = 0.100; Figure 4). Several males did not emit food calls at all (n = 47). The average FOH‐score was significantly lower in the males that did emit at least one food call (n = 16), compared to those that emitted none (t[63] = −11.43, *p* < 0.001; Figure 4). Neither latency to reach the food zone nor latency to food peck were affected by FOH‐score in any of the sexes (χ^2^_1_ = 2.335, *p* > 0.05 vs. χ^2^_1_ = 0.149, *p* > 0.05; Figure 4).

There were no other significant effects of FOH‐score on the other measured variables in the behaviour tests (Supplementary Table S4).

### 3.2 Body weight

**FIGURE 5:**
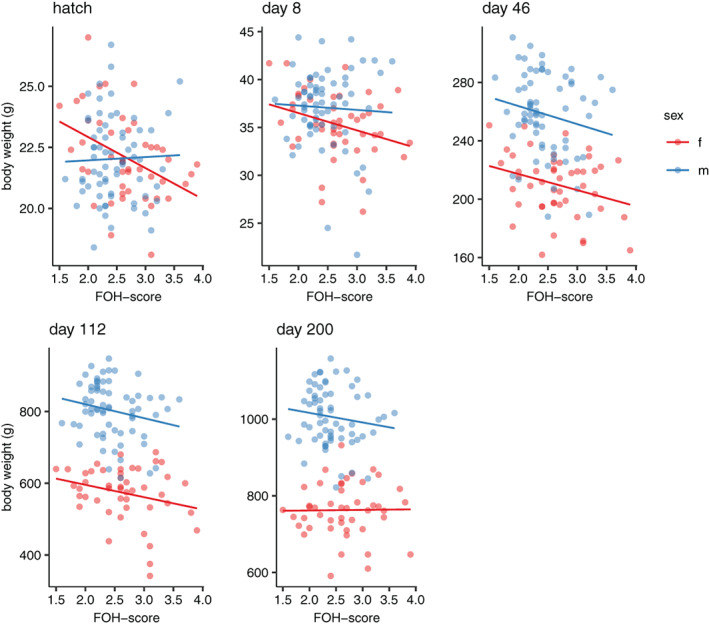
Scatterplots of body weight at different ages plotted against fear‐of‐human (FOH) score in males and females

**FIGURE 6:**
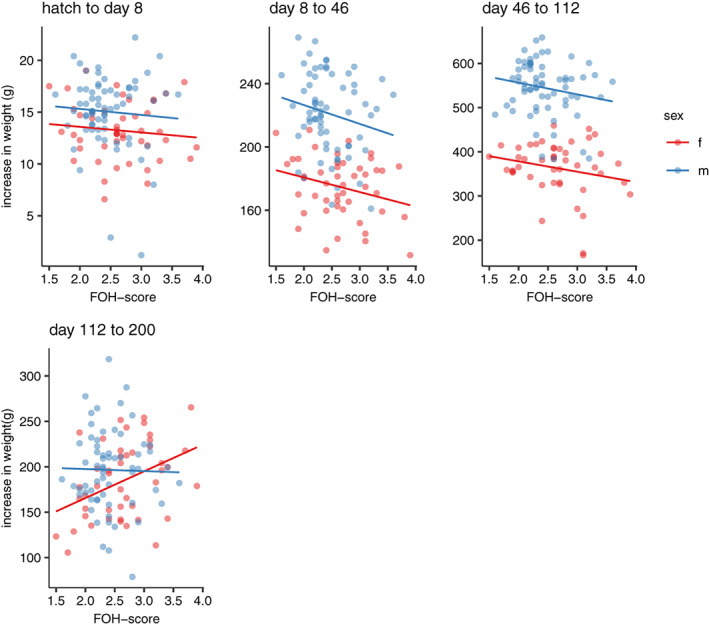
Scatterplots of growth rate in different age‐intervals. Weight gain plotted against fear‐of‐human (FOH) score in males and females

There was a significant effect of the FOH score of the parents (in the F2‐generation) and the hatch weight of the F3 chicks, where offspring of parents with higher FOH score were overall heavier at hatch (Spearman correlation test, females: r_s_ = 0.35, *p* < 0.001; males: r_s_ = 0.39, *p* < 0.001). In the F3 birds, lower FOH‐score in females was associated with higher hatch weight, with a significant interaction effect between FOH‐score and sex (χ^2^_1_ = 4.428, *p* = 0.035; Figure 5). FOH‐score also had a significant effect on body weight at 46 (χ^2^_1_ = 5.489, p = 0.019) and 112 days of age (χ^2^_1_ = 5.781, *p* = 0.016), where higher body weight was associated with lower FOH‐score (Figure 5). Furthermore, FOH‐score had a significant effect on weight gain, both between 8 and 46 days (χ^2^_1_ = 5.517, *p* = 0.019) as well as between 46 and 112 days of age (χ^2^_1_ = 4.002, *p* = 0.045), with lower FOH‐score being associated with higher weight gain (Figure 5). There was also a significant effect of the interaction between FOH‐score and sex on the weight gain between 112 and 200 days of age (χ^2^_1_ = 3.929, *p* = 0.047) where females with higher FOH‐scores caught up in growth relative to those with lower FOH‐scores (Figure 6), which could explain the lack of effect of FOH‐score on body weight at day 200 (Supplementary Table S1).

### 3.3 Brain size

As expected, given the large difference in body size between the sexes (Figure 5), all absolute brain weight measurements differed significantly between males and females (Supplementary Table S2). Body weight and ROB significantly affected absolute brain and brain regions weight both for males and females (Supplementary Table S3).

**FIGURE 7:**
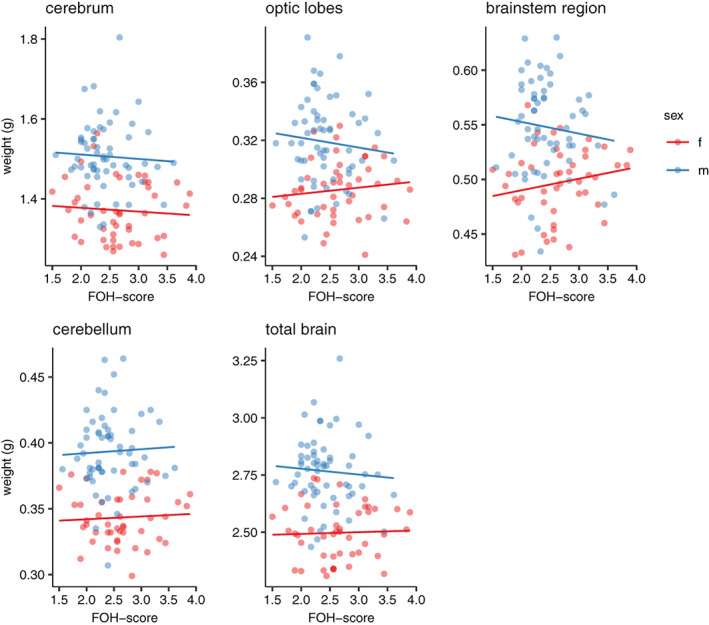
Scatterplots of absolute brain region weights as well as total brain weight plotted against fear‐of‐human (FOH) score in males and females

In females, for absolute cerebra and brainstem region weights there was a tendency for an effect of FOH‐score (cerebrum χ^2^_1_ = 3.622, *p* = 0.057; brainstem region χ^2^_1_ = 3.141, 0.076). Females with a lower FOH‐score tended to have larger cerebra, but smaller brainstem regions (Figure 7). There were no effects of FOH‐score for any other regions in females and neither the separate regions, nor absolute brain weight, were affected by FOH‐score in the males (Supplementary Table S3, Figure 7).

**FIGURE 8:**
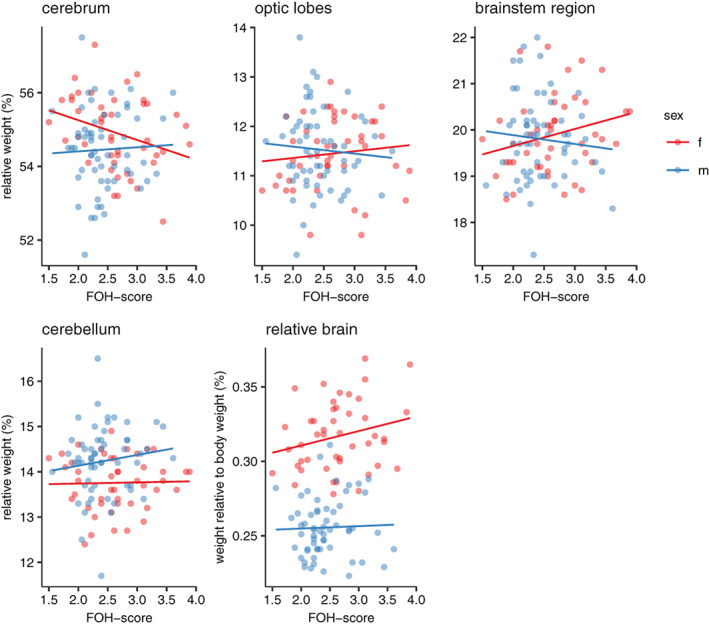
Scatterplots of brain region weight relative to rest of brain as well as total brain weight relative to body weight plotted against fear‐of‐human (FOH) score in males and females

Relative cerebrum size was significantly affected by FOH‐score in females (χ^2^_1_ = 4.786, *p* < 0.05), where the relative cerebrum weight was higher in females with lower FOH‐score. Furthermore, there was a tendency towards an effect of FOH‐score on both relative brainstem region weight (χ^2^_1_ = 3.338, *p* = 0.068) as well as brain weight relative to body weight (χ^2^_1_ = 2.789, *p* = 0.095) in females. Both brainstem region and relative brain size were lower in females with lower FOH‐score (Figure 8). FOH‐score had no effect on the remaining brain regions in females and had no effect on any regions, or relative brain size in males (Supplementary Table S3, Figure 8).

## 4 DISCUSSION

To shed light on the role of tameness in the evolution of domesticated phenotypes in chickens, we studied an F3‐intercross between two lines of Red Junglefowl, previously selected during eight generations for divergent scores in a “fear‐of‐humans (FOH) test”. We found that body weight and growth rate was correlated with FOH‐score, in that less fearful birds were bigger and grew faster. Low FOH‐score in females was also correlated with increased activity in an open field test and faster fear habituation, and in males it was correlated with increased tendency to emit food calls (indicating increased social dominance) in a mirror‐based social encounter test. Furthermore, in females, low FOH‐score was associated with increased relative size of cerebrum and a tendency for reduced relative brain stem size as well as reduced brain size relative to body weight. The results concur with the effects previously reported in comparisons between ancestral Red Junglefowl and less fearful domesticated laying hens.^18^, ^22^ This indicates that tameness may drive at least some aspects of the domestication syndrome in chickens through either pleiotropy or linkage.

When comparing the distributions of the FOH‐scores in the parental generation and the F3 intercross, it is clear that the FOH‐score follows a normal distribution in the F3, which is what is expected when crossing two lines that are diverging in a continuous trait. This indicates an additive polygenic inheritance of the FOH‐scores, consistent with the relatively low, although significant, heritability of 0.17 that was found in the second selected generation.^9^ In the present study, we have further analysed which traits that are correlated to FOH‐score in the F3‐intercross, and that may thus be either genetically linked to FOH‐score or affected by pleiotropy.

We found that lower FOH‐score was associated with higher body weight and faster growth, at least before puberty. Previous studies of the selection lines have shown that chickens from the low fear line are larger than the high fear line chickens,^11^ suggesting that body size changes as a correlated response to selection for tameness. Low fear line chickens also have a higher basal metabolic rate and greater feed efficiency.^23^ This is consistent with earlier comparisons between Red Junglefowl and domesticated White Leghorn layers that reported QTLs, which simultaneously affect both fear related behaviour and growth.^24^

Our present results suggest that body weight is genetically affected by loci related to tameness, either through linkage or pleiotropy, and this could be a contributing factor to the large difference between wild Red Junglefowl (adult weight about 800–1000 g) and domesticated breeds (often about twice this size^25^). Determining the exact mechanisms of the correlated selection responses will require in‐depth genetic analyses. In this study, we have only tested correlations between phenotypes, and the next logical step would be to conduct a QTL analysis to find genetic regions of interest that could be affecting the different traits in the particular intercross we have studied.

A possible hypothesis that should be tested in such in‐depth genetic analysis could be that reduced stress sensitivity, associated with tameness, reduces the activity in the hypothalamus‐pituitary–adrenal (HPA) axis, which in turn may facilitate a higher growth rate.^26^ In White Leghorn laying hens, pituitary expression of genes that are key inhibitors of the stress response is higher than in Red Junglefowl,^27^ consistent with the hypothesis that tameness, stress and growth may be intimately connected. Furthermore, a non‐significant, but numerically higher, HPA‐responsiveness has previously been demonstrated in a small sample of females from the high fear selection line, compared to the low fear line, again consistent with the hypothesis.^23^

We found several effects of FOH‐score on brain size and composition in the females. Brain size relative to body weight tended to be smaller in females with lower FOH‐score, and cerebrum size was significantly larger relative to the size of the total brain. Both relative and absolute brainstem size tended to be smaller and absolute cerebrum size tended to be larger, going in the same direction as that for the relative size. In previous studies, we similarly found that absolute and relative brain size differed between the selection lines and that these changes are probably due to size changes in specific brain regions as opposed to a general effect on the brain as a whole.^28^ Similar effects were found in a comparison between Red Junglefowl and White Leghorns,^18^ showing that selection responses can act independently on separate brain regions. Overall, we show that selection on tameness changes brain composition, consistent with known domestication effects, where domesticated animals generally evolve smaller brains, possibly as a result of reallocating resources to more energy demanding traits.^29^

Previously, chickens from the low fear selection line have been found to be less fearful in the open field test^9^ although this has not been consistent in successive generations.^30^ Here, we found that females with lower FOH‐scores moved longer distances in the open field. In chickens, high activity is generally accepted as the main indicator of low fearfulness in the open field test.^31^ The results therefore show that reduced FOH‐score is related to a general reduction in fearfulness, possibly again supporting the idea that tameness may affect other behaviour via modifications of the HPA‐axis.

Similarly, the fear habituation test showed that females with a lower FOH‐score had a significantly smaller AUC on test day 2, which means that their reaction on the second exposure to the test was less fearful. This indicates that they have a better long‐term habituation to artificial frightening stimuli such as a sudden flash of light. Our observation here corresponds to earlier findings in our selection lines, where chickens from the low fear line were better at habituating to the artificial stimulus (Katajamaa and Jensen, in press). While selection on tameness does not seem to generalise to all other traits related to fearfulness,^30^ we do find effects on open field and fear habituation behaviours, showing that these may be genetically linked to FOH‐score.

Earlier studies on the selection lines have found that chickens from the low fear line are more dominant when tested in different competitive situations against size matched chickens from the high selection line.^11^ Since the present study required a standardised value of social dominance for each bird, a new test was designed that did not require confrontation with a real opponent. We used a mirror that creates an image of a matched opponent of the same sex, and placed the food source close to the mirror so that it would appear that a food competition event arose whenever the tested bird attempted to feed. Our expectation was that more dominant birds would show aggressive behaviour against the mirror image, but that was only observed in a few instances. However, we did find that males with a lower FOH‐score tended to emit more food calls and to do so with a shorter latency. Food calls are emitted mainly by males and its main function is to attract females.^32^ This behaviour is strongly correlated with social dominance, and typically males who emit frequent food calls are preferred by females for mating.^33^ Although we did not observe much obvious aggressive behaviour, the results still indicate that social dominance in males may be linked to low FOH‐score.

Since we have done an intercross between two divergently selected lines, the idea is that traits that correlate do so because they are affected by pleiotropic or linked genes. Intercrosses like this have proven useful for studying the genetic causes underlying differences between domesticated and wild populations of several species.^34^ The exact mechanisms of these correlated selection responses cannot be determined through the experimental design used here. For this, genetic mapping using QTL‐analysis, and subsequent gene expression analysis can be a fruitful way forward. For example, by overlaying QTL‐regions with expression‐QTLs in an intercross between Red Junglefowl and White Leghorn laying hens, five strong candidate genes involved in the domestication of social behaviour in chickens have been identified.^35^ Similar approaches will be the next logical step in further studies of the present intercross population.

It is clear that, overall, the effects of FOH‐scores on other traits measured in this study were affected by interaction effects with sex. This means that either one of the sexes was affected or that they were affected in different directions. Interaction effects between sex and selection line or breed are not uncommon, and we have seen this type of effects before both in our selection lines as well as in comparisons between Red Junglefowl and domesticated breeds.^24^, ^30^ This may be a result of different genetic architectures in the two sexes and corroborates previous findings showing large sex differences in behaviour in chickens.^36^ Furthermore, the correlations between FOH‐scores and other traits are relatively weak, which indicates that many genes are involved in the traits measured here, corroborating previous genetic mapping in crosses between Red Junglefowl and domesticated birds.^35^

Or results support the general hypothesis that early domestication could have been driven to a large extent by intentional or unintentional selection on tameness. As a result, phenotypes correlated to the domesticated phenotype could have emerged as correlated selection responses.^6^, ^28^ We have previously shown that FOH in ancestral Red Junglefowl has a genetic component and can be selected upon to breed considerably tamer birds in few generations, which simultaneously develop traits commonly associated with the domesticated phenotype.^9^ In this study, we further show that some of the traits are likely to be pleiotropic or linked to FOH. This is consistent with the hypothesis that tameness may have driven the evolution of domesticated phenotypes in chickens.

In conclusion, in an intercross between lines of Red Junglefowl selected for divergent levels of the selection variable FOH‐score, lower FOH‐score was correlated to larger body size and increased growth. Furthermore, low FOH‐score in females was correlated with increased activity in an open field test and more efficient fear habituation, and in males it was correlated with increased tendency to emit food calls in a social encounter test, indicating higher social dominance. With respect to brain size, in females lower FOH‐score was associated with reduced relative brain size, increased relative cerebrum size, and reduced relative brainstem region. For most of the traits, the effect of FOH‐score interacted with sex, leading to absent or opposite effects in one of the sexes. These effects are consistent with those observed in domesticated chickens, so the results indicate that tameness may drive at least some aspects of the domestication syndrome through either pleiotropy or linkage. For future research, the genetic relationship between tameness and other traits should be investigated by more rigorous genetic analyses, such as QTL studies.

## CONFLICT OF INTEREST

The authors have no conflicts of interest to declare.

## ETHICS STATEMENT

All experimental protocols were approved by Linköping Council for Ethical Licencing of Animal Experiments, ethical permits no 50–13, and 14,916–2018. Experiments were carried out in accordance with the approved guidelines.

## Supporting information


**Appendix S1**: Supporting information

## Data Availability

The data that supports the findings of this study are available in the supplementary material of this article
