# Supplementary material for: Tameness correlates with domestication related traits in a Red Junglefowl intercross
Source: Genes Brain Behav. 2020 Oct 12;20(3):e12704. doi: 10.1111/gbb.12704 (PMC7988571; doi:10.1111/gbb.12704)
Supplement: Supplementary file 1 — Appendix S1: Supporting information [file GBB-20-e12704-s001.docx]

# Supplementary information

**Table S1.** Results from generalized linear model for body weight and growth rate with phenotype against fear score and sex.

*Significant effect

#Tendency towards significant, 0.05 < p < 0.1

| **Phenotype** | **Model term** | **χ^2^_1_** | **p-value** |
| --- | --- | --- | --- |
| **Body weight** |  |  |  |
| Hatch | sex* | 4.752 | .029 |
|  | FOH-score# | 3.087 | .079 |
|  | sex: FOH-score* | 4.428 | .035 |
| Day 8 | sex | .285 | .593 |
|  | FOH-score | 2.295 | .130 |
|  | sex: FOH-score | .903 | .342 |
| Day 46 | sex# | 3.590 | .058 |
|  | FOH-score* | 5.489 | .019 |
|  | sex: FOH-score | .004 | .947 |
| Day 112 | sex* | 9.026 | .003 |
|  | FOH-score* | 5.781 | .016 |
|  | sex: FOH-score | .005 | .943 |
| Day 200 | sex* | 16.536 | .000 |
|  | FOH-score | .629 | .428 |
|  | sex: FOH-score | .808 | .369 |
| **Growth rate** |  |  |  |
| Hatch to day 8 | sex | .249 | .617 |
|  | FOH-score | .736 | .391 |
|  | sex: FOH-score | .001 | .981 |
| Day 8 to 46 | sex* | 4.825 | .028 |
|  | FOH-score* | 5.517 | .019 |
|  | sex: FOH-score | .055 | .815 |
| Day 46 to 112 | sex* | 8.521 | .004 |
|  | FOH-score* | 4.002 | .045 |
|  | sex: FOH-score | .004 | .951 |
| Day 112 to 200 | sex* | 5.294 | .021 |
|  | FOH-score | 2.391 | .122 |
|  | sex: FOH-score* | 3.929 | .047 |

**Table S2**. Means (± standard error) and results from T-test performed on differences in body- and brain size between the sexes. Relative weights for brain regions are relative to total brain weight.

*Significant difference between males and females

| **Test variable** | **females** | **males** | **test statistic (t)** | **df** | **p-value** |
| --- | --- | --- | --- | --- | --- |
| **Absolute weights** |  |  |  |  |  |
| Body weight* | 791.9 (± 9.1) | 1087 (± 10.4) | -21.318 | 102.999 | <0.001 |
| Cerebrum* | 1.37 (± 0.0110) | 1.51 (± 0.0861) | -8.586 | 101.865 | <0.001 |
| Optic lobes* | 0.286 (± 0.00296) | 0.319 (± 0.00382) | -6.843 | 101.408 | <0.001 |
| Brainstem region* | 0.497 (± 0.00484) | 0.548 (± 0.00555) | -6.948 | 102.990 | <0.001 |
| Cerebellum* | 0.343 (± 0.00289) | 0.393 (± 0.00377) | -10.559 | 101.195 | <0.001 |
| Total brain* | 2.50 (± 0.0175) | 2.77 (± 0.0201) | -10.092 | 102.981 | <0.001 |

**Table S3.** Results from generalized linear model for measures of absolute and relative brain weights.

*Significant effect

#Tendency towards significant, 0.05 < p < 0.1

|  |  | **Females** |  | **Males** |  |  |
| --- | --- | --- | --- | --- | --- | --- |
|  | **Model term** | **χ^2^_1_** | **p-value** | **χ^2^_1_** | **p-value** |  |
| **Absolute brain** |  |  |  |  |  |  |
| Cerebrum | FOH-score | 3.622# | 0.057 | 0.000 | 0.985 |  |
|  | ROB cerebrum | 47.064* | <0.001 | 69.718* | <0.001 |  |
| Optic lobes | FOH-score | 0.694 | 0.405 | 0.353 | 0.553 |  |
|  | ROB optic lobes | 5.480* | 0.019 | 22.319* | <0.001 |  |
| Brainstem region | FOH-score | 3.141# | 0.076 | 0.548 | 0.459 |  |
|  | ROB brainstem region | 33.774* | <0.001 | 49.183* | <0.001 |  |
| Cerebellum | FOH-score | 0.138 | 0.711 | 0.797 | 0.372 |  |
|  | ROB cerebellum | 21.416* | <0.001 | 29.893* | <0.001 |  |
| Total brain | FOH-score | 0.867 | 0.352 | 0.096 | 0.756 |  |
|  | body weight | 11.638* | 0.001 | 7.949* | 0.005 |  |
| **Relative brain** |  |  |  |  |  |  |
| Cerebrum | FOH-score | 4.786* | 0.029 | 0.077 | 0.781 |  |
| Optic lobes | FOH-score | 0.513 | 0.474 | 0.323 | 0.570 |  |
| Midbrain | FOH-score | 3.338# | 0.068 | 0.470 | 0.493 |  |
| Cerebellum | FOH-score | 0.046 | 0.831 | 1.068 | 0.301 |  |
| Relative brain | FOH-score | 2.789# | 0.095 | 0.080 | 0.777 |  |

**Table S4.** Results from generalized linear model for behaviours measured in the fear habituation, open field and mirror tests. Spearman rank correlation test results reported for frequency of food calls and crowing in males.

*Significant effect/correlation

#Tendency towards significant, 0.05 < p < 0.1

| **Behaviour** | **Model term** | **χ^2^_1_** | **p-value** |
| --- | --- | --- | --- |
| **Fear habituation test** |  |  |  |
| Fear reaction day 1 | sex | 0.053 | 0.819 |
|  | FOH-score | 0.531 | 0.466 |
|  | sex: FOH-core | 0.126 | 0.722 |
| Fear reaction day 2 | sex# | 3.263 | 0.071 |
|  | FOH-score | 0.096 | 0.757 |
|  | sex: FOH-score* | 4.306 | 0.038 |
| **Open field test** |  |  |  |
| Time spent in centre | sex | 1.097 | .295 |
|  | FOH-score | .011 | .917 |
|  | sex: FOH-score | 1.391 | .238 |
| Distance moved | sex* | 7.384 | .000 |
|  | FOH-score | .017 | .896 |
|  | sex: FOH-score* | 6.003 | .014 |
| **Mirror test** |  |  |  |
| Latency food peck (container) | sex | .082 | .775 |
|  | FOH-score | .149 | .699 |
|  | sex: FOH-score | .000 | .986 |
| Latency food zone | sex | .078 | .781 |
|  | FOH-score | 2.335 | .126 |
|  | sex: FOH-score | .255 | .613 |
| Latency food call males | FOH-score# | 2.708 | .100 |
| **Mirror test**  *Spearman rank correlation* |  | **r_S_** | **p-value** |
| Frequency food call males | FOH-score* | -.334 | .007 |
| Frequency crowing males | FOH-score | -.061 | .635 |
